# Physical, Cognitive and Emotional Factors Contributing to Quality of Life, Functional Health and Participation in Community Dwelling in Chronic Kidney Disease

**DOI:** 10.1371/journal.pone.0091176

**Published:** 2014-03-10

**Authors:** Ulla K. Seidel, Janine Gronewold, Michaela Volsek, Olga Todica, Andreas Kribben, Heike Bruck, Dirk M. Hermann

**Affiliations:** 1 Department of Neurology, University Hospital Essen, Essen, Germany; 2 Department of Nephrology, University Hospital Essen, Essen, Germany; Julius-Maximilians-Universität Würzburg, Germany

## Abstract

**Background:**

Quality of life (QoL) impairment is a well-known consequence of chronic kidney disease (CKD). The factors influencing QoL and late life functional health are poorly examined.

**Methods:**

Using questionnaires combined with neuropsychological examinations, we prospectively evaluated physical, cognitive, and emotional factors influencing QoL, functional health and participation in community dwelling in 119 patients with CKD stages 3–5 including hemodialysis (61.5±15.7years; 63% men) and 54 control patients of the same age without CKD but with similar cardiovascular risk profile.

**Results:**

Compared with control patients, CKD patients showed impairment of the physical component of QoL and overall function, assessed by the SF-36 and LLFDI, whereas disability, assessed by LLFDI, was selectively impaired in CKD patients on hemodialysis. Multivariable linear regressions (forced entry) confirmed earlier findings that CKD stage (β = −0.24; p = 0.012) and depression (β = −0.30; p = 0.009) predicted the QoL physical component. Hitherto unknown, CKD stage (β = −0.23; p = 0.007), cognition (β = 0.20; p = 0.018), and depression (β = −0.51; <0.001) predicted disability assessed by the LLFDI, while age (β = −0.20; p = 0.023), male gender (B = 5.01; p = 0.004), CKD stage (β = −0.23; p = 0.005), stroke history (B = −9.00; p = 0.034), and depression (β = −0.41; p<0.001) predicted overall function. Interestingly, functional health deficits, cognitive disturbances, depression, and anxiety were evident almost only in CKD patients with coronary heart disease (found in 34.2% of CKD patients). The physical component of QoL and functional health decreased with age and depressive symptoms, and increased with cognitive abilities.

**Conclusions:**

In CKD, QoL, functional health, and participation in community dwelling are influenced by physical, cognitive, and emotional factors, most prominently in coronary heart disease patients.

## Introduction

Chronic kidney disease (CKD) is a worldwide growing health problem that is found in 23–35% of adults above 64 years [1]. Quality of life (QoL) is reduced in this population [2]. The underlying reasons are largely unknown. A better understanding is important, because QoL is closely linked to patient morbidity and mortality [3]. QoL is also frequently used as outcome measure in CKD treatment studies [3]. In CKD patients requiring dialysis, QoL has previously been associated with compliance to dialysis, hospitalization and survival [4]–[6].

CKD patients exhibit high loads of vascular risk factors and co-morbidities [7]–[9], compromising QoL in this patient group [10]. As a matter of fact, vascular diseases are not the only factors influencing QoL in CKD: CKD patients frequently present with cognitive deficits and depressive symptoms [9]. Depressive symptoms have frequently been studied in stage 5D patients on dialysis, where they predisposed to QoL impairment [11]–[14], but less often in earlier stages of CKD [15]–[17]. Whether and how cognitive performance affects QoL in CKD was hitherto unknown.

For the evaluation of late life functioning, assessments of functional health and participation in community dwelling are widely used in health services research [18], [19]. In age-related diseases, the Late Life Function and Disability Instrument (LLFDI), which consists of a disability and overall function dimension that can be broken down into various subscales, is particularly well validated [20], [21]. Together with the Medical Outcomes Study Short Form-36 (SF-36) health survey [22] and a detailed neuropsychological examination, this tool allows particularly thorough QoL investigations.

Considering the lack of a comprehensive study that examined the effect of physical, cognitive and emotional factors on QoL, functional health and participation in community dwelling, we prospectively studied 119 patients with CKD stages 3–5 and 5D, which we compared with a control group consisting of 54 control patients without CKD, but with similar age and vascular risk profile. In these subjects, we investigated QoL and late life functional health using the SF-36 and LLFDI questionnaires, and performed a detailed neuropsychological examination, in which we evaluated the patients' cognitive performance.

## Methods

### Ethics Statement

This study was approved by the ethics committee of the faculty of medicine at the University of Duisburg-Essen (08-3817). All participants enrolled in the study provided written informed consent.

### Participants

The New Tools for the Prevention of Cardiovascular Disease in CKD study (NTCVD) is a prospective study analyzing the effects of risk factors and markers of cardiovascular disease in patients with CKD [23]. 119 patients with CKD stages 3–5 (according to K/DOQI-classification) [24] including hemodialysis were recruited together with 54 control patients with similar cardiovascular risk profile but without CKD at the Department of Nephrology at the University Hospital Essen with the help of local physicians. 30 of 119 patients had CKD stage 3A (45≤estimated glomerular filtration rate [eGFR]<60 ml/min/1.73m^2^), 21 had CKD stage 3B (30≤eGFR<45 ml/min/1.73m^2^), 21 had CKD stage 4 (15≤eGFR<30 ml/min/1.73m^2^), 12 had CKD stage 5 not requiring hemodialysis (eGFR<15 ml/min/1.73m^2^) and 35 had CKD stage 5D requiring hemodialysis (any eGFR). All patients underwent a standardized interview to collect demographic data and a medical history, followed by a physical examination, laboratory analyses and neuropsychological testing. All patients completed standardized questionnaires to assess quality of life, functional health and participation in community dwelling, and mood disturbances.

### Medical History and Laboratory Tests

The detailed medical history included information about the onset and treatment of CKD, known cardiovascular risk factors, associated illnesses (specifically coronary heart disease (CHD), myocardial infarct, stroke, transient ischemic attack (TIA), peripheral artery disease (PAD)), and current medications. Blood and urine samples were collected, in which the stage of CKD and vascular risk profile were evaluated in addition to routine blood parameters. eGFR was calculated using the modification of diet in renal disease formula (MDRD). Education was assessed by evaluating the highest academic degree and patient years of school education.

### Standardized Questionnaires

Three standardized self-report questionnaires were completed:

1. QoL was assessed using the SF-36 health survey [22]. The SF-36 consists of two summary scores, the physical component score and mental component score, that are broken down in eight subscales (‘physical function’, ‘physical role’, ‘bodily pain’, ’general health perceptions’, ‘mental health’, ’emotional role’, ‘social function’, ’vitality’). Raw values of all summary scales were transformed into standardized 0–100 scales. Higher scores indicate better QoL or less impairment for each domain.
2. Functional health and participation in community dwelling was evaluated by the LLFDI [20], [21]. The LLFDI consists of a total disability component score, composed of two summary scales, the disability dimension and overall function. The disability dimension is broken down in frequency of performance (measuring social and personal role domains) and limitation in capability (measuring instrumental and management role domains). The function component assesses overall function in three subcategories (upper extremity, basic lower extremity and advanced lower extremity). All items are scored on a 5-point ordinal scale. Component scores range from 0–100, higher scores indicating less impairment. Raw scores were transferred in scaled scores by summing up subscale relevant items.
3. Depression and anxiety were examined using the Hospital Anxiety and Depression Scale (HADS) [25]. The HADS is a 14-item questionnaire, of which 7 items each examine depressive and anxiety symptoms on a 4-point scale. As such, scores from 0–21 points are obtained for both subscales, higher scores indicating higher symptom prevalence. In accordance with Zigmond and Snaith (1983) [25], categories were formed with 0–7 points being interpreted as normal, 8–10 points as borderline finding, and ≥11 points as suggestive for depression or anxiety.

### Neuropsychological Assessment

Participants were examined with a battery of ten standardized tests, namely (a) the revised Wechsler memory scale (subtests digit and block span, forward and backward; assessment of verbal and spatial short-term and working memory) [26], (b) the trail making test (TMT) part A (assessment of information processing speed) [27], (c) the trail making test part B (assessment of executive flexibility) [27], (d) the Stroop test (assessment of executive flexibility) [28], (e) the Regensburg word fluency test (subtests ‘animals’ and ‘s-words’; assessment of lexical and semantical word fluency) [29] and (f) the Rey-Osterrieth complex figure test (copy task, assessment of visuo-constructive function) [30]. For CKD patients, z-scores were calculated for all ten neuropsychological tests based on norm values generated in the control cohort [9]. A global cognitive performance variable was formed by computing mean values of these z-scores. This global cognitive performance variable was used for further analyses. To rule out major confounders of cognitive performance as a consequence of patient tremor, the Whiget tremor rating scale was used. Examiners were not notified about the medical condition of the participants by the recruiting staff.

### Statistical Analysis

Continuous data are expressed as mean±SD for normally and median (Q1;Q3) for non-normally distributed variables, categorical data as counts (%). For continuous data, group differences between control patients and all CKD patients were calculated with unpaired two-tailed t-tests (normally distributed data) or Mann-Whitney tests (non-normally distributed data). To compare control patients with CKD patients with or without dialysis, (a) one-way analyses of variance (ANOVA) followed by Bonferroni (equal variances) or Games-Howell (unequal variances) post-hoc tests (normally distributed continuous data) and (b) Kruskall-Wallis tests followed by Mann-Whitney tests with Bonferroni corrections for multiple testing (non-normally distributed continuous data) were used. Categorical data were evaluated by chi-square or Fisher tests. Predictors of QoL, functional health and participation in community dwelling, and depression were analyzed using univariate and multivariable linear regressions (forced entry strategy). In the regression studies, SD values were used as unit for determining β values. All comparisons were performed with SPSS 19 for Windows (SPSS Inc., Chicago, IL).

## Results

### Study Cohort

The characteristics of the NTCVD cohort are summarized in Table 1. CKD patients in this study had a mean age of 61.5±15.7 years, with a predominance of men (63%) in the sample. As described before [9], the age and gender profile did not differ from control patients. Compared with patients belonging to CKD stages 3–5 that did not receive renal replacement therapy, patients with CKD stage 5D on hemodialysis were younger and slightly (though not significantly) more often men. Education assessed as highest academic degree did not differ between groups, although CKD stage 3–5 patients not requiring dialysis had significantly less years of school education than control patients. Handedness did not differ between groups. CKD patients showed higher Whiget tremor scores for the left hand (median (Q1;Q3) = 0 (0;2)) compared with control patients (0 (0;0)).

**Table 1:** Characteristics of control patients and CKD patients, the latter also split into patients not requiring and requiring hemodialysis.

	Controls(n = 54)	CKD all(all, n = 119)	CKD stages 3–5(n = 84)	CKD stage 5D(n = 35)
**Age (years)**	62.3±10.1	61.5±15.7	64.5±13.5	54.6±18.4†
**Gender (male)**	40 (74.1)	75 (63.0)	48 (57.1)	27 (77.1)
**Highest academic degree**				
No degree	1 (1.9)	10 (8.4)	8 (9.5)	2 (5.7)
Secondary School	40 (74.1)	94 (79.0)	66 (78.6)	28 (80.0)
Baccalaureate	7 (13.0)	9 (7.6)	5 (6.0)	4 (11.4)
University degree	6 (11.1)	6 (5.0)	5 (6.0)	1 (2.9)
**Education (years of school)**	10 (8;10)	9 (8;10)*	8 (8;10)*	9 (8;10)
**Handedness (right hand)**	45 (83.3)	99 (83.2)	67 (79.8)	32 (91.4)
**Tremor right hand (score)**	0 (0;0)	0 (0;1)	0 (0;1)	0 (0;1)
**Tremor left hand (score)**	0 (0;0)	0 (0;2)*	0 (0;2)*	0 (0;3)*
**CHD/myocardial infarct**	22 (40.7)	40 (34.2)	25 (30.5)	15 (42.9)
**PAD**	2 (3.7)	12 (10.2)	7 (8.4)	5 (14.3)
**Stroke/TIA**	0	7 (5.9)*	7 (8.4)	0†
**Hemoglobin (g/dl)**	14.1±1.3	12.5±1.7*	12.5±1.6*	12.5±1.9*
**Creatinine (mg/dl)**	1.1±0.2	3.6±2.7*	2.5±1.9*	6.2±2.3* †
**eGFR (ml/min/1.73m^2^)**	70.9±9.6	28.2±17.6*	35.2±16.0*	n.a.
**Time since first dialysis (months)**	n.a.	n.a	n.a.	21.0 (5.3;42.4)
**Time since last dialysis (hours)**	n.a.	n.a	n.a.	28.0 (22.5;35.5)
**Cognitive Performance (mean z-scores)**	0.00±0.58	−0.53±0.84*	−0.56±0.81*	−0.46±0.90*
**Cognitive Impairment (more than 1 z-score below controls)**	Reference	36 (30.3)*	26 (31.0)*	10 (28.6)*
**HADS-depression scale (mean score)**	5.0 (2.0;6.5)	4.0 (2.0;8.0)	4.0 (2.0;7.0)	6.0 (2.0;13.0)
**HADS-depression scale (score ≥11)**	3 (5.6)	18 (15.1)	7 (8.3)	11 (31.4)* †
**HADS-anxiety scale (mean score)**	5.0 (3.0;8.0)	5.0 (2.0;7.0)	5.0 (2.0;7.0)	6.0 (4.0;12.0)
**HADS-anxiety scale (score ≥11)**	7 (13.0)	19 (16.0)	10 (11.9)	9 (25.7)

Data are means ± SD for normally distributed continuous data or median (Q1;Q3) for non-normally distributed continuous data, categorical data are presented as n (%). Continuous data were evaluated by unpaired two-tailed t-tests for comparisons between control patients and all CKD patients. Continuous data were evaluated by oneway ANOVA followed by Bonferroni or Games-Howell (in case of non-equal variances) tests (normally distributed data) or Kruskall-Wallis followed by Mann-Whitney tests (non-normally distributed data) for comparisons between control patients, CKD patients requiring hemodialysis and CKD patients not requiring hemodialysis. Categorical data were evaluated by chi-square or Fisher exact tests.

*p<0.05 compared with control patients,

† p<0.05 compared with CKD patients not requiring hemodialysis. CHD, coronary heart disease; CKD, chronic kidney disease; eGFR, estimated glomerular filtration rate; ESRD, end stage renal disease; HADS, Hospital Anxiety and Depression Scale; PAD, peripheral artery disease; TIA, transient ischemic attack.

### Medical History and Laboratory Findings

Medical history and laboratory findings are also shown in Table 1. As expected, CKD patients exhibited a high prevalence of CHD or myocardial infarcts (34.2%) and PAD (10.2%). Only 5.9% reported strokes or TIAs. The prevalence of cardiovascular diseases did not differ between control patients, CKD stage 3–5 patients not receiving hemodialysis, and CKD stage 5D patients receiving hemodialysis. Stroke prevalence was higher in CKD stage 3–5 patients (8.4%) than in control (0%) and stage 5D (0%) patients. Hemoglobin (Hb) was lower in CKD stage 3–5 (12.5±1.7 g/dl) and stage 5D (12.5±1.6 g/dl) patients than in control patients (14.1±1.3 g/dl). Creatinine was elevated in CKD, more strongly in stage 5D (median (Q1;Q3) = 5.9 (4.4;8.1) mg/dl) than stage 3–5 (1.8 (1.3;3.2) mg/dl; control patients: 1.1 (0.9;1.2) mg/dl). Mean eGFR in the CKD group not requiring dialysis was 35.2±16.0 ml/min/1.73 m^2^, compared with 70.9±9.6 ml/min/1.73 m^2^ in control patients.

### Cognition and Mood

Neuropsychological test results, depression and anxiety scores are also shown in Table 1. As previously shown [9], global cognitive performance, evaluated by z-scores as mean of ten neuropsychological tests, was worse in CKD stage 3–5 (−0.56±0.81) and stage 5D (−0.46±0.90) than in control (0.0±0.58) patients. 30.3% of CKD patients had a poor cognitive performance that was more than one standard deviation below controls.

Mean depression and anxiety scores, evaluated by HADS, did not significantly differ between control patients and CKD groups. Yet, stage 5D patients receiving hemodialysis had a higher prevalence of depressive symptoms (31.4% with HADS score≥11), than control patients (5.6%) and CKD stage 3–5 patients not receiving hemodialysis (8.3%). The prevalence of anxiety symptoms did not significantly differ between groups.

### Quality of Life

In the SF-36 questionnaire, stage 3–5 (40.7±11.3) and stage 5D (40.5±10.3) patients showed a significant lower physical component score than control patients (47.4±11.2) (Table 2). The mental component score did not differ between groups. In the physical component, CKD patients showed impairments in physical function, physical role and general health. In the mental component, CKD patients revealed impairments in social function. In the latter score, stage 5D patients had significant lower values than stage 3–5 patients not requiring dialysis.

**Table 2:** Quality of life data of control subjects and CKD patients, the latter also split into patients not requiring and requiring hemodialysis.

	Controls(n = 54)	CKD all(n = 119)	CKD stages 3–5 (n = 84)	CKD stage 5D (n = 35)
**SF-36: Physical Component**	47.4±11.2	40.6±11.0*	40.7±11.3*	40.5±10.3*
SF-36: Physical Function	90.0 (70.0;96.3)	67.5 (41.9;90.0)*	67.5 (40.0;90.0)*	62.5 (45.0;90.0)*
SF-36: Role Physical	100.0 (50.0;100.0)	75.0 (0;100.0)*	75.0 (0;100.0)*	62.5 (0;100.0)*
SF-36: Bodily Pain	82.0 (41.0;100.0)	74.0 (41.0;100.0)	74.0 (51.0;100)	67.0 (31.0;100.0)
SF-36: General Health	61.0±22.9	46.3±19.9*	48.4±18.5*	41.2±22.3*
**SF-36: Mental Component**	49.9±10.7	49.4±11.0	51.1±9.3	45.3±13.6
SF-36: Mental Health	76.0 (59.0;88.0)	73.5 (55.6;88.0)	76.0 (57.0;88.0)	68.0 (36.0;84.0)
SF-36: Role Emotional	100.0 (66.7;100.0)	100.0 (66.7;100.0)	100.0 (66.7;100.0)	100.0 (0;100.0)
SF-36: Social Function	100.0 (75.0;100.0)	87.5 (56.3;100.0)*	87.5 (62.5;100.0)*	62.5 (50.0;100.0)* †
SF-36: Vitality	58.7±17.7	52.7±21.4	54.2±20.1	48.9±24.1
**LLFDI: Disability Component**	135.2±13.6	121.4±21.4*	122.9±20.1*	118.3±24.0*
**LLFDI: Disability Dimension**	67.0±7.5	62.3±9.9*	63.5±9.3	59.7±10.9*
LLFDI: Frequency of Performance	30.5±4.0	28.7±5.0*	29.1±4.8	27.6±5.1*
LLFDI: Social Role Domain	13.2±2.7	12.2±3.1	12.3±3.1	12.1±3.0
LLFDI: Personal Role Domain	17.3±2.2	16.5±2.9*	16.9±2.8	15.5±2.9* †
LLFDI: Limitation in Capability	38.0 (35.0;40.0)	36.0 (29.0;40.0)*	36.0 (30.0;40.0)	34.5 (27.0;37.8)*
LLFDI: Instrumental Role Domain	19.0 (17.5;20.0)	18.0 (15.0;20.0)*	18.0 (14.5;20.0)	17.0 (15.0;20.0)
LLFDI: Management Role Domain	19.0 (19.0;20.0)	18.0 (15.0;20.0)*	19.0 (16.0;20.0)*	17.2 (12.3;20.0)*
**LLFDI: Overall Function**	68.3±8.3	60.1±12.8*	60.9±12.0*	58.2±14.6*
LLFDI: Upper Extremity	25.0 (24.0;25.0)	24.0 (20.0;25.0)*	24.0 (21.0;25.0)*	23.0 (19.0;25.0)*
LLFDI: Basic Lower Extremity	29.0 (27.0;30.0)	27.0 (22;30.0)*	27.0 (23.0;30.0)*	26.0 (20.0;30.0)*
LLFDI: Advanced Lower Extremity	17.0 (14.0;19.0)	14.0 (9.0;18.0)*	14.0 (9.0;18.0)*	14.0 (8.0;18.0)*

Data are means ± SD for normally distributed data or median (Q1;Q3) for non-normally distributed data. Data were evaluated by unpaired two-tailed t-tests for comparisons between control patients and all CKD patients. Data were evaluated by oneway ANOVA followed by Bonferroni or Games-Howell (non-equal variances) tests (normally distributed data) or Kruskall-Wallis followed by Mann-Whitney tests (non-normally distributed data) for comparisons between control subjects, CKD patients requiring hemodialysis and CKD patients not requiring hemodialysis.

*p<0.05 compared with control subjects,

† p<0.05 compared with CKD patients not requiring hemodialysis. CKD, chronic kidney disease; ESRD, end stage renal disease; LLFDI, Late-Life Function and Disability Instrument; SF-36, 36-Item Short Form Health Survey.

### Functional Health and Participation in Community Dwelling

CKD patients had worse scores in the LLFDI disability dimension (62.3±9.9) and overall function (60.1±12.8) scales than control patients (disability dimension: 67.0±7.5, overall function: 68.3±8.3), which was evident in all subscales except in the social role domain (Table 2). In general, CKD stage 3–5 patients not on hemodialysis did not differ from stage 5D patients on hemodialysis, except for the personal role domain, in which stage 5D patients exhibited lower scores.

### Predictors for Quality of Life: Role of Medical Conditions

To evaluate QoL predictors, univariate and multivariable linear regressions were computed for the whole cohort using a variety of models in which demographic factors (age, gender, education), CKD-related factors (CKD stage, hemoglobin), vascular diseases (CHD/myocardial infarct, stroke/TIA, PAD), cognitive performance, depression and anxiety were inserted in different combinations (Table 3). In univariate regressions, the factors age (β = −0.22; p = 0.005), education (β = 0.18; p = 0.020), CKD stage (β = −0.21; p = 0.006), CHD/myocardial infarct (B = −3.83; p = 0.041), PAD (B = −7.22; p = 0.030), cognitive performance (β = 0.26; p = 0.001) depression (β = −0.36; p<0.001) and anxiety (β = −0.21; p = 0.001) were predictors of the physical component of QoL, while the factors CHD/myocardial infarct (B = −4.12; p = 0.020), depression (β = −0.62; p<0.001) and anxiety (β = −0.68 per SD; p<0.001) were predictors of the mental component of QoL.

**Table 3:** Predictors for Quality of life in all patients: Role of medical conditions.

	Physical Component								Mental Component							
	Univariate		Model 1		Model 2		Model 3		Univariate		Model 1		Model 2		Model 3	
	B or β	p	B or β	p	B or β	p	B or β	p	B or β	p	B or β	p	B or β	p	B or β	p
**Age**	−0.22	**0.005**	−0.29	**0.001**	−0.23	**0.018**	−0.17	0.100	0.04	0.639	0.03	0.736	0.05	0.624	0.07	0.387
**Gender (male vs. female)**	0.22	0.910	1.06	0.574	1.66	0.410	2.84	0.151	−1.65	0.369	−1.04	0.588	−0.21	0.918	0.79	0.589
**Education**	0.18	**0.020**	0.08	0.292	0.04	0.650	0.84	0.401	0.03	0.746	0.04	0.674	0.04	0.677	−0.07	0.295
**CKD stage**	−0.21	**0.006**	−0.31	**0.001**	−0.30	**0.002**	−0.24	**0.012**	−0.10	0.237	−0.13	0.184	−0.01	0.344	0.02	0.818
**Hemoglobin**	0.10	0.217	0.01	0.951	0.01	0.961	−0.01	0.974	−0.04	0.659	−0.09	0.324	−0.06	0.544	0.00	0.978
**CHD/myocardial infarct (yes vs. no)**	3.83	**0.041**	–	–	3.53	0.089	1.83	0.369	4.12	**0.020**	–	–	3,87	0.066	0.75	0.619
**Stroke/TIA (yes vs. no)**	7.18	0.104	–	–	4.26	0.340	3.39	0.434	0.15	0.971	–	–	1,32	0.770	0.12	0.969
**PAD (yes vs. no)**	7.22	**0.030**	–	–	1.61	0.649	1.28	0.705	−2.36	0.456	–	–	−3,48	0.332	−3.69	0.143
**Cognitive performance**	0.26	**0.001**	–	–	–	–	0.06	0.558	0.15	0.065	–	–	–	–	0.09	0.214
**HADS-depression scale**	−0.36	**<0.001**	–	–	–	–	−0.30	**0.009**	−0.62	**<0.001**	–	–	–	–	−0.35	**<0.001**
**HADS-anxiety scale**	−0.21	**<0.001**	–	–	–	–	0.01	0.922	−0.68	**<0.001**	–	–	–	–	−0.45	**<0.001**

In multivariable regressions (forced entry strategy), the physical component of QoL was predicted by the factors age (β = −0.23; p = 0.018) and CKD stage (β = −0.30; p = 0.002) (model 2 in Table 3, which contains demographic factors, CKD-related factors and vascular diseases), the latter of which disappeared, while depression became predictive (β = −0.30; p = 0.009), when cognitive performance, depression and anxiety were inserted into the regression analysis (full model 3 in Table 3). The mental component of QoL was predicted by the factors depression (β = −0.35; p<0.001) and anxiety (β = −0.45; p<0.001) only (full model 3 in Table 3).

### Predictors of Functional Health and Participation in Community Dwelling: Role of Medical Conditions

Since impairments in daily life activities have an impact on QoL in other medical conditions [31], we next examined predictors of functional health and participation in community dwelling using the LLFDI disability dimension and overall function summary scores with the same univariate and multivariable linear regression models as above (Table 4). In univariate regressions, the factors age (β = −0.22; p = 0.007), male gender (B = −3.85; p = 0.018), CKD stage (β = −0.27; p = 0.001), CHD/myocardial infarct (B = −4.46; p = 0.007), cognitive performance (β = 0.39; p<0.001), depression (β = −0.58; p<0.001) and anxiety (β = −0.33; p<0.001) predicted the disability dimension of the LLFDI (in which higher scores indicate better health), whereas the factors age (β = −0.29; p<0.001), education (β = 0.16; p = 0.037), CKD stage (β = −0.25; p = 0.001), hemoglobin (β = 0.17; p = 0.025), CHD/myocardial infarct (B = −5.78; p = 0.002), stroke/TIA (B = −12.79; p = 0.012), PAD (B = −9.27; p = 0.007), cognitive performance (β = 0.36; p<0.001), depression (β = −0.48; p<0.001) and anxiety (β = −0.26; p = 0.001) predicted overall function.

**Table 4:** Predictors for functional health and participation in community dwelling in all patients: Role of medical conditions.

	Disability Dimension										Overall Function									
	Univariate		Model 1		Model 2			Model 3			Univariate		Model 1		Model 2			Model 3		
	B or β	p	B or β	p		B or β	p		B or β	p	B or β	p	B or β	p		B or β	p		B or β	p
**Age**	−0.22	**0.007**	−0.31	**<0.001**		−0.22	**0.021**		−0.07	0.414	−0.29	**<0.001**	−0.39	**<0.001**		−0.29	**0.001**		−0.20	**0.023**
**Gender (male vs. female)**	−3.85	**0.018**	−2.94	0.062		−2.76	0.084		−1,30	0.340	1.29	0.515	2.18	0.244		3.77	**0.047**		5,01	**0.004**
**Education**	0.09	0.276	−0.03	0.711		−0.06	0.453		−0.07	0.388	0.16	**0.037**	0.02	0.763		−0.01	0.920		−0.01	0.873
**CKD stage**	−0.27	**0.001**	−0.34	**<0.001**		−0.37	**<0.001**		−0.23	**0.007**	−0.25	**0.001**	−0.31	**<0.001**		−0.32	**<0.001**		−0.23	**0.005**
**Hemoglobin**	0.10	0.223	0.01	0.909		−0.01	0.956		−0.01	0.941	0.17	**0.025**	0.08	0.313		0.04	0.635		0.03	0.714
**CHD/myocardial infarct (yes vs. no)**	−4.46	**0.005**	–	–		−4.04	**0.018**		−1,81	0.144	−5.78	**0.002**	–	–		−5.54	**0.005**		−3,12	0.089
**Stroke/TIA (no vs. yes)**	7.65	0.054	–	–		−6.62	0.081		−5,78	0.071	12.79	**0.012**	–	–		−9.45	**0.044**		−9,00	**0.034**
**PAD (yes vs. no)**	−2.81	0.295	–	–		3.51	0.200		3,37	0.214	−9.27	**0.007**	–	–		−1.90	0.572		−2,06	0.499
**Cognitive performance**	0.39	**<0.001**	–	–		–	–		0.20	**0.018**	0.36	**<0.001**	–	–		–	–		0.11	0.168
**HADS-depression scale**	−0.58	**<0.001**	–	–		–	–		−0.51	**<0.001**	−0.48	**<0.001**	–	–		–	–		−0.41	**<0.001**
**HADS-anxiety scale**	−0.33	**<0.001**	–	–		–	–		0.05	0.635	−0.26	**0.001**	–	–		–	–		0.02	0.821

For the adjusted models, linear regressions were calculated using a forced entry strategy. Model 1: Age, gender, education, CKD stage, hemoglobin. Model 2: Age, gender, education, CKD stage, hemoglobin, CHD or myocardial infarct, stroke or TIA, PAD. Model 3: Age, gender, education, CKD stage, hemoglobin, CHD or myocardial infarct, stroke or TIA, PAD, global cognitive performance, depression and anxiety. CHD, coronary heart disease; CKD, chronic kidney disease; HADS, Hospital Anxiety and Depression Scale; LLFDI, Late-Life Function and Disability Instrument; PAD, peripheral artery disease; SF-36, 36-Item Short Form Health Survey.

In multivariable regressions, the factors age (β = −0.22 per SD; p = 0.021), CKD stage (β = −0.37; p<0.001) and CHD/myocardial infarct (B = −4.04; p = 0.018) predicted the LLFDI disability dimension, whereas age (β = −0.29; p = 0.001), male gender (B = 3.77; p = 0.047), CKD stage (β = −0.32; p<0.001), CHD/myocardial infarct (B = −5.54; p = 0.005) and stroke/TIA (B = −9.45; p = 0.044) predicted overall function (model 2 in Table 4, which contains demographic factors, CKD-related factors and vascular diseases). When cognitive performance, depression and anxiety were included into multivariable regression analyses (model 3 in Table 4), CHD/myocardial infarct did not predict the disability dimension and overall function any more. Instead, cognitive performance (β = 0.20; p = 0.018) and depression (β = −0.51; p<0.001) (disability dimension) or depression (β = −0.41; p<0.001) (overall function) became predictive.

### Predictors of Quality of Life in CKD: Role of Functional Health and Participation in Community Dwelling

To evaluate how functional health and participation in community dwelling influenced QoL in CKD patients, univariate and multivariable linear regressions were again evaluated, in which demographic factors, CKD-related factors (both as above) and LLFDI scores were inserted (Table 5). In univariate regressions, the factors age (β = −0.22; p = 0.005), CKD stage (β = −0.21; p = 0.006), disability dimension (β = 0.56; p<0.001), overall function (β = 0.80; p<0.001), cognitive performance (β = 0.26; p<0.001), depression (β = −0.38 per SD; p<0.001) and anxiety (β = −0.21; p = 0.008) predicted the physical component of QoL that was assessed by the SF-36, whereas the factors disability dimension (β = 0.35; p<0.001), overall function (β = 0.21; p = 0.001), depression (β = −0.62; p<0.001) and anxiety (β = −0.68; p<0.001) predicted the mental component of QoL.

**Table 5:** Predictors for quality of life in all patients: Role of functional health and participation in community dwelling.

	Physical Component								Mental Component							
	Univariate		Model 1		Model 2		Model 3		Univariate		Model 1		Model 2		Model 3	
	B or β	p	B or β	p	B or β	p	B or β	p	B or β	p	B or β	p	B or β	p	B or β	p
**Age**	−0.22	**0.005**	−0.03	0.699	−0.20	**0.031**	−0.04	0.472	0.04	0.639	0.105	0.200	0.13	0.111	0.07	0.368
**Gender (male vs. female)**	0.22	0.910	−0.02	0.988	3.60	0.057	0.37	0.752	−1.65	0.369	0.935	0.530	0.10	0.946	1.13	0.431
**Education**	0.18	0.202	0.09	0.127	0.12	0.127	0.09	0.081	0.03	0.746	−0.081	0.232	−0.09	0.187	−0.10	0.138
**CKD stage**	−0.21	**0.006**	−0.08	0.152	−0.17	**0.027**	−0.07	0.145	−0.09	0.237	0.032	0.642	0.04	0.561	0.01	0.845
**LLFDI:Disability Dimension**	0.56	**<0.001**	0.05	0.545	–	–	–	–	0.35	**<0.001**	0.049	0.592	–	–	–	–
** LLFDI:Frequency of Performance**	0.37	**<0.001**	–	–	0.19	**0.050**	–	–	0.28	**<0.001**	–	–	−0.05	0.573	–	–
** LLFDI:Limitation in Capability**	0.55	**<0.001**	–	–	0.34	**<0.001**	–	–	0.31	**<0.001**	–	–	0.04	0.640	–	–
**LLFDI:Overall Function**	0.80	**<0.001**	0.78	**<0.001**	–	–	–	–	0.21	**0.001**	−0.094	0.279	–	–	–	–
** LLFDI:Upper Extremity**	0.53	**<0.001**	–	–	–	–	−0.07	0.288	0.19	**0.016**	–	–	–	–	−0.10	0.245
** LLFDI:Basic Lower Extremity**	0.76	**<0.001**	–	–	–	–	0.42	**<0.001**	0.19	**0.014**	–	–	–	–	0.02	0.881

For the adjusted models, linear regressions were calculated using a forced entry strategy. Model 1: Age, gender, education, CKD stage, Disability Dimension, Overall Function, depression, anxiety and global cognitive performance. Model 2: Age, gender, education, CKD stage, Frequency of Performance, Limitation in Capability, depression, anxiety and global cognitive performance 3: Age, gender, education, CKD stage, Upper Extremity, Basic Lower Extremity, Advanced Lower Extremity, depression, anxiety and global cognitive performance. CKD, chronic kidney disease; HADS, Hospital Anxiety and Depression Scale; LLFD, Late-Life Function and Disability Instrument; SF-36, 36-Item Short Form Health Survey.

In multivariable regressions, the factor overall function (β = 0.78; p<0.001) was the only predictor of the physical component of QoL, while the factors cognitive performance (β = 0.17; p = 0.035), depression (β = −0.34; p = 0.001) and anxiety (β = 0.44; p<0.001) predicted the mental component (model 1 in Table 5). When inserting subscales of overall function into the regression analysis, basic lower extremity (β = 0.42; p<0.001) and advanced lower extremity (β = 0.50; p<0.001) function predicted the physical component (model 3 in Table 5).

### Predictors of Depressive Symptoms in CKD: Role of Medical Conditions

In view of the eminent effect of depression on QoL in CKD, we subsequently analyzed predictors of depressive symptoms in CKD patients using univariate and multivariable linear regressions, in which demographic factors, CKD-related factors, vascular diseases and cognitive performance (all as above) were inserted (Table 6). In univariate regressions, the factors CKD stage (β = 0.18; p = 0.017), CHD/myocardial infarct (B = 2.22; p = 0.001) and cognitive performance (β = −0.19; p = 0.012) were predictors of depressive symptoms as evaluated by the HADS depression scale, whereas in multivariable regressions the factor CHD/myocardial infarct alone (B = 1.77; p = 0.018) predicted depressive symptoms (full model 3 in Table 6).

**Table 6:** Disease relevant predictors for depressive symptoms in all patients.

	HADS-depression scale							
	Univariat		Model 1		Model 2		Model 3	
	B or β	p	B or β	p	B or β	p	B or β	p
**Age**	0.10	0.188	0.16	0.068	0.11	0.267	0.06	0.548
**Gender (male vs. female)**	1.28	0.050	1.06	0.115	0.79	0.264	0.68	0.341
**Education**	−0.04	0.621	0.01	0.869	0.03	0.708	0.04	0.688
**CKD stage**	0.18	**0.017**	0.22	**0.013**	0.18	0.061	0.153	0.114
**Hemoglobin**	−0.05	0.511	0.00	0.960	−0.01	0.267	−0.01	0.958
**CHD/myocardial infarct (yes vs. no)**	−2.22	**0.001**	–	–	−1.89	**0.011**	−1.77	**0.018**
**Stroke/TIA (yes vs. no)**	−0.01	0.903	–	–	−0.21	0.897	0.02	0.990
**PAD (yes vs. no)**	−1.17	0.303	–	–	0.01	0.992	−0.01	0.995
**Cognitive Performance**	−0.19	**0.012**	–	–	–	–	−0.10	0.328

For the adjusted models, linear regressions were calculated using a forced entry strategy. Model 1: Age, gender, education, CKD stage, hemoglobin. Model 2: Age, gender, education CKD stage, hemoglobin, CHD or myocardial infarct, stroke or TIA, PAD. Model 3: Age, gender, education, CKD stage, CHD or myocardial infarct, stroke or TIA, PAD, global cognitive performance. CHD, coronary heart disease; CKD, chronic kidney disease; HADS, Hospital Anxiety and Depression Scale; PAD, peripheral artery disease; TIA, transient ischemic attack.

### Predictors of Depressive Symptoms in CKD: Role of Functional Health and Participation in Community Dwelling

To evaluate how functional health and participation in community dwelling affect depression in CKD, we finally analyzed predictors of depressive symptoms using univariate and multivariable linear regressions, in which demographic factors, CKD-related factors and LLFDI scores (all as above) were included (Table 7). In univariate regressions, the factors CKD stage (β = 0.18 per SD; p = 0.017), disability dimension (β = −0.58; p<0.001), overall function (β = −0.50; p<0.001) and cognitive performance (β = −0.10; p = 0.012) predicted depressive symptoms, whereas in multivariable regressions, the factors disability dimension (β = −0.45; p<0.001), overall function (β = −0.21; p = 0.036) and cognitive performance (β = 0.05; p = 0.036) predicted depressive symptoms (model 1 in Table 7). Further analyses revealed that both frequency of performance (β = −0.33; p<0.001) and limitation in capability (β = −0.35; p<0.001) influenced depressive symptoms within the disability dimension (model 2 in Table 7), whereas upper extremity function (β = −0.22; p = 0.025) influenced depressive symptoms in the overall function dimension (model 3 in Table 7).

**Table 7:** Predictors for depressive symptoms in all patients: Role of functional health and participation in community dwelling.

HADS-depression scale								
	Univariate		Model 1		Model 2		Model 3	
	B or β	p	B or β	p	B or β	p	B or β	p
**Age**	0.10	0.188	−0.05	0.584	−0.00	0.986	−0.05	0.617
**Gender (male vs. female)**	1.28	0.050	0.71	0.267	0.23	0.742	1.61	**0.010**
**Education**	−0.04	0.621	−0.01	0.863	−0.02	0.764	0.01	0.855
**CKD stage**	0.18	**0.017**	−0.01	0.949	0.02	0.804	0.04	0.656
**LLFDI:Disability Dimension**	−0.58	**<0.001**	−0.45	**<0.001**	–	–	–	–
LLFDI:Frequency of Performance	−0.48	**<0.001**	–	–	−0.33	**<0.001**	–	–
LLFDI:Limitation in Capability	−0.48	**<0.001**	–	–	−0.35	**<0.001**	–	–
**LLFDI:Overall Function**	−0.50	**<0.001**	−0.21	**0.036**	–	–	–	–
LLFDI:Upper Extremity	−0.41	**<0.001**	–	–	–	–	−0.22	**0.025**
LLFDI:Basic Lower Extremity	−0.46	**<0.001**	–	–	–	–	−0.17	0.190
LLFDI:Advanced Lower Extremity	−0.41	**<0.001**	–	–	–	–	−0.16	0.149
**Global cognitive performance**	−0.10	**0.012**	0.05	**0.036**	0.05	0.553	−0.02	0.836

For the adjusted models, linear regressions were calculated using a forced entry strategy. Model 1: Age, gender, education, CKD stage, Disability Dimension, Overall Function and global cognitive performance. Model 2: Age, gender, education, CKD stage, Frequency of Performance, Limitation in Capability and global cognitive performance. Model 3: Age, gender, education, CKD stage, Upper Extremity, Basic Lower Extremity, Advanced Lower Extremity and global cognitive performance. CKD, chronic kidney disease; HADS, Hospital Anxiety and Depression Scale; LLFD, Late-Life Function and Disability Instrument.

### Effect of Age, Cognition and Depression on Quality of Life and Related Outcome Measures

**Figure 1:**
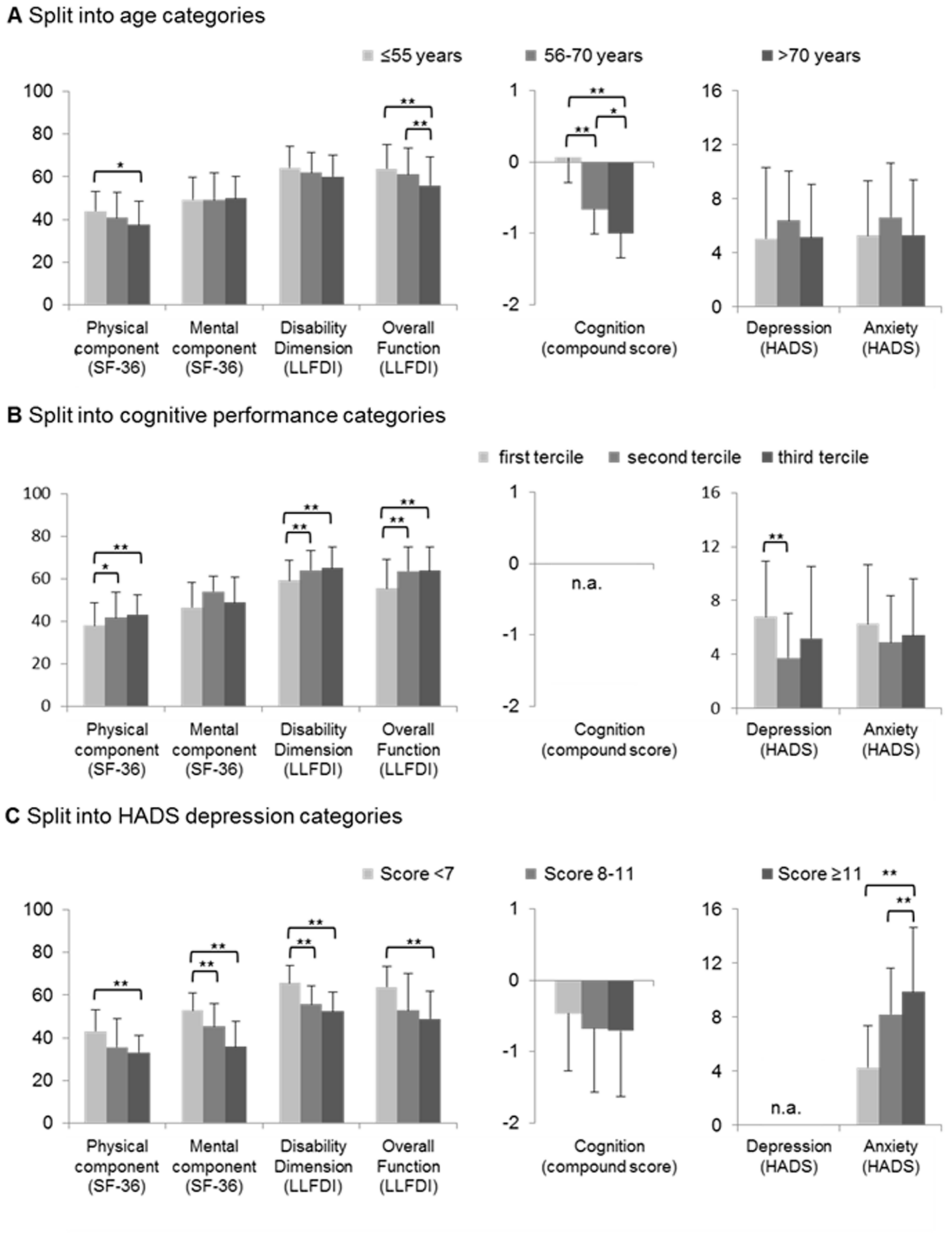
Quality of life, functional health and participation in community dwelling, cognition, depression and anxiety: Role of age, cognition and depressive symptoms. Note that (A) QoL, functional health and participation in community dwelling, and cognitive performance decrease with age. (B) Patients with higher cognitive performance show better physical abilities. Patients with (C) higher depression scores show impairment in QoL and functional health. Data are means and standard deviations. Significance was evaluated by one-way ANOVA followed by Bonferroni (equal variances) or Games-Howell (non-equal variances) tests (in case of normally distributed data) or Kruskall-Wallis followed by Mann-Whitney tests with Bonferroni corrections (in case of non-normally distributed data). *p<0.05/**p<0.01. Cognitive compound score represents z-scores (calculated based on norm values generated in the control cohort), which cover ten neuropsychological tests. First tercile: <−0.620 z-scores, second tercile: −0.620≤×<−0.016 z-scores, third tercile: ≥−0.016 z-scores. CKD, chronic kidney disease; HADS, Hospital Anxiety and Depression Scale; LLFDI, Late-Life Function and Disability Instrument; SF-36, 36-Item Short Form Health Survey.

To elucidate how age, cognition and depression influenced quality of life and related outcome measures, we stratified the data based on age categories (≤55 years, 55<x≤70 years, >70 years), cognitive performance terciles (<−0.620 z-scores, −0.620≤×<−0.016 z-scores, ≥−0.016 z-scores) and HADS depression categories (<7 points, 7≤×<11 points, ≥11 points). These analyses showed that the physical component of QoL, overall function of the LLFDI and cognitive performance decreased with increasing age, while the mental component of QoL, the disability dimension of the LLFDI, depression and anxiety did not change with age (Fig. 1A). The physical component of QoL, the disability dimension of the LLFDI and the overall function dimension of the LLFDI increased with cognitive capabilities, whereas depression scores were higher in subjects with low than intermediate cognitive function (Fig. 1B). Both the physical and mental component of QoL and the disability and overall function dimension of the LLFDI decreased with increasing depression scores, as did anxiety (Fig. 1C).

### Effect of CHD/myocardial Infarcts on Quality of Life and Related Outcome Measures

**Figure 2:**
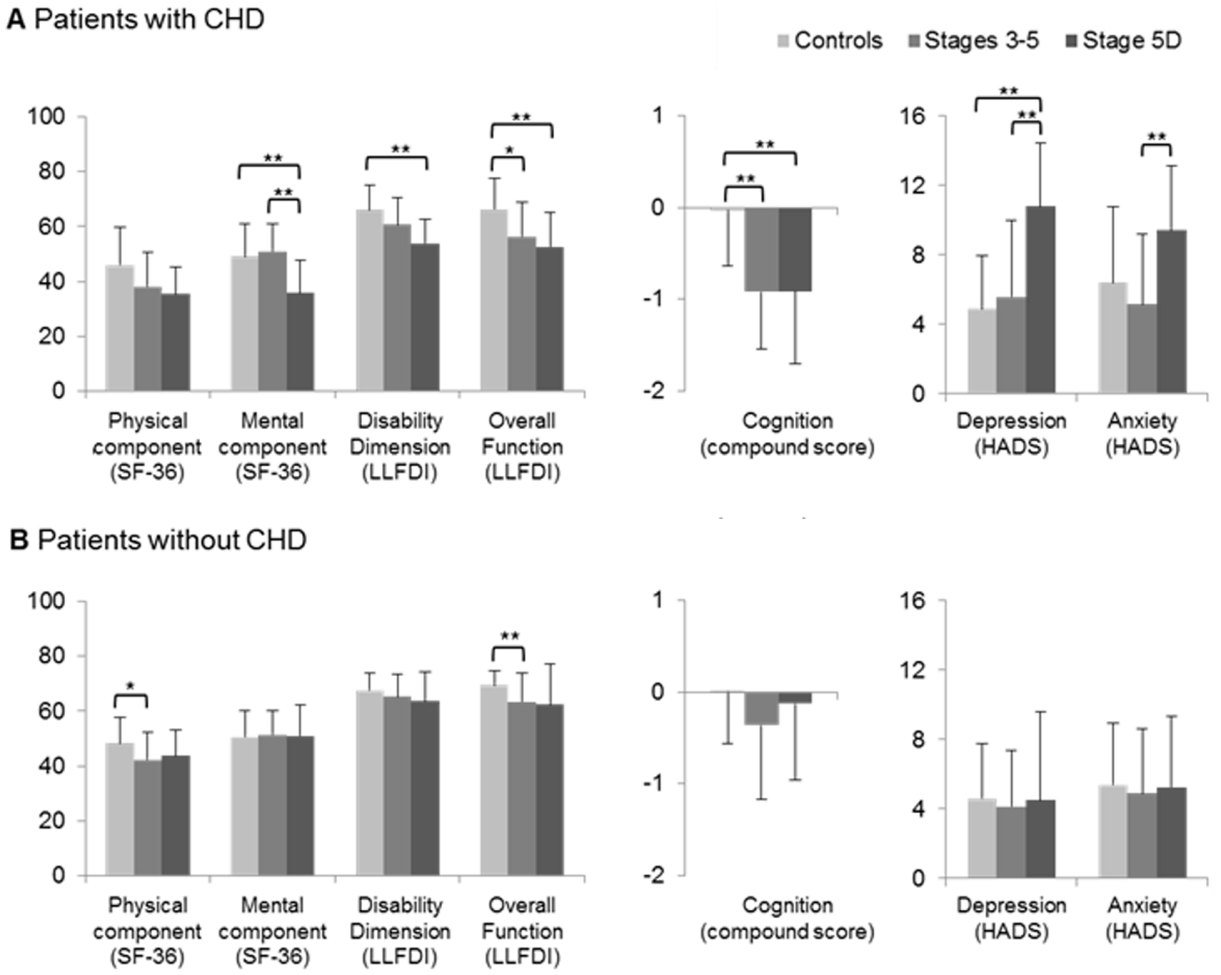
Quality of life, functional health and participation in community dwelling, cognition, depression and anxiety: Role of CKD and CHD. Note that compared with control patients, CKD patients with CHD show impairment in all domains except the physical component of the SF-36. Such impairment is almost absent in patients without CHD. Data are means and standard deviations. Significance was evaluated by one-way ANOVA followed by Bonferroni (equal variances) or Games-Howell (non-equal variances) tests (in case of normally distributed data) or Kruskall-Wallis followed by Mann-Whitney tests with Bonferroni corrections (in case of non-normally distributed data). *p<0.05/**p<0.01. Cognitive compound score represents z-scores (calculated based on norm values generated in the control cohort), which cover ten neuropsychological tests. CHD, coronary heart disease; CKD, chronic kidney disease; HADS, Hospital Anxiety and Depression Scale; LLFDI, Late-Life Function and Disability Instrument; SF-36, 36-Item Short Form Health Survey.

To elucidate how the presence of CHD or myocardial infarcts modified quality of life related outcome measures, we compared patients with and without CHD/myocardial infarcts in their history. These studies showed that while the mental component of QoL, the disability dimension of the LLFDI, the overall function dimension of the LLFDI and cognition were reduced in CKD patients with CHD/myocardial infarcts (most strongly and in case of the disability dimension significant only in CKD stage 5D patients), depression was increased in stage 5D patients with CHD/myocardial infarcts (Fig. 2A). Notably, the latter relationships were not at all noticed in CKD patients not exhibiting CHD/myocardial infarcts in their history (Fig. 2B). These CKD patients did not reveal a reduced mental component of QoL or a reduced disability dimension of the LLFDI, and they did not exhibit an impaired cognitive performance or an increased depression score.

## Discussion

We for the first time provide a comprehensive analysis of physical, cognitive and emotional factors influencing QoL, functional health and participation in community dwelling using a cohort of 119 patients with CKD stages 3–5 (including stage 5D requiring hemodialysis) that we compared with 54 control patients of the same age with similar cardiovascular risk profile without CKD. As such, we confirmed previous findings that the physical component of QoL, which is impaired both in CKD stage 3–5 and stage 5D patients, is predicted by CKD stage and depressive symptoms, furthermore showing that the disability dimension of functional health and participation in community dwelling, which assesses socially defined life tasks as part of the LLFDI, is in addition influenced by cognitive performance. Age, male gender, CKD stage, stroke history and depression are predictors of overall function, which measures the ability to perform discrete actions and activities without assistance within the LLFDI. Functional health deficits, cognitive and emotional disturbances were found mainly in CKD patients suffering from CHD, thus further corroborating the close relationship between physical factors, cognition and emotion and health-related QoL in CKD.

Our observation that QoL is reduced in the physical, but not mental domain, is well in line with previous studies [11], [13], [15], [16], [32]–[34]. Interestingly, stage 3–5 and stage 5D patients did not differ to major extent in the physical component of QoL and the overall function dimension of functional health and participation in community dwelling. In case of QoL assessments, patients with advanced (stages 4 and 5) CKD not requiring dialysis previously exhibited either slightly better [15] or comparable [32] QoL scores than stage 5D patients on dialysis. Differences in the constitution of CKD cohorts and dialysis protocols may account for diverging findings in the past. Systematic analyses of functional health and participation in community dwelling have not yet been performed in CKD patients. For assessments of daily life activities, the Karnofsky score has mainly been used in the past [11], [34]. The Karnofsky score has been developed for the evaluation of chemotherapies in oncology patients [35]. Unlike the LLFDI, the Karnofsky score represents a rough scale of independence stages ranging from 0 to 100% that does not provide more differentiated insights into late life function and disabilities.

Linear regressions revealed that CHD and myocardial infarcts were the most important predictors both for the disability dimension and overall function dimension of the LLFDI in addition to age and CKD stage, when age, gender, education, CKD stage, hemoglobin and vascular diseases were inserted into a multivariable analysis. Interestingly, CHD and myocardial infarcts did not continue to be predictive for both functional health scales any more, when cognitive performance, depression and anxiety were included into the regression analyses. Thus, depression and - in case of the disability dimension - cognition and anxiety were identified as predictors of functional health and participation in community dwelling. That CHD and myocardial infarcts indeed potently influenced functional health in CKD was confirmed in subsequent studies showing that the mental component of QoL, the disability dimension of the LLFDI, overall function of the LLFDI and cognitive performance decreased with progressive CKD severity in patients suffering from CHD, but not in those without. Moreover, depression and anxiety were selectively increased in CKD stage 5D patients suffering from CHD. That cardiovascular diseases are major factors influencing QoL in CKD has previously been shown [24], [36]. In addition to earlier studies, we herein demonstrate that CHD modifies cognitive performance, depression and anxiety in CKD patients. We have previously reported that the inflammation marker fibrinogen predicts cognitive performance in CKD patients in multivariable regression analyses [9]. Whether inflammation associated with CHD is responsible for CKD-related QoL and functional health deficits remains to be shown.

Our observation of a link between CHD, functional health and QoL argues in favor of the so-called ‘concept of loss’, upon which the loss of physical strength and social activities contributes to depression that in turn compromises QoL [37]. In that study, patients with CKD were characterized using a so-called Kidney Disease Loss Scale that aimed to identify the most severe individual-defined losses and subsequently evaluated grief responses associated with these losses. That depression is highly prevalent in CKD and that it contributes to QoL deficits, is well established [32], [37]–[40]. Although the link between physical deficits and depression is plausible, assessments of overall function in our study revealed that upper extremity deficits were particularly associated with depressive symptoms, whereas lower extremity deficits were associated with reduced QoL. Our observations suggest that physical conditions may affect depression and QoL in different ways.

It is known that the physical component of QoL decreases with age [32], [33], [41], [42] and depressive symptoms [32], [36] in CKD. In line with these findings, our studies revealed that the QoL physical component and overall function of the LLFDI decreased with age and depressive symptoms. Interestingly and hitherto unknown, not only age and depressive symptoms, but also cognitive performance were found to modify health-related QoL. Thus, the physical component of QoL, the disability dimension of the LLFDI and the overall function dimension of the LLFDI increased with cognitive performance. An obvious strength of this study is the recruitment of an own control group of patients without CKD with similar cardiovascular risk profile. In fact, all except three [10], [43], [44] previous studies used healthy control subjects for evaluating QoL in CKD. Given that this cross-sectional study establishes associations and not causal relationships, longitudinal studies are urgently needed for evaluating the role of physical, cognitive and emotional factors in CKD.
